# Could elective nodal irradiation for locally advanced rectal cancer be omitted in the context of total neoadjuvant therapy? An analysis of the recurrence sites of rectal cancer

**DOI:** 10.3389/fonc.2024.1459024

**Published:** 2024-11-27

**Authors:** Linlin Xiao, Shiyu Zhuo, Yuanhang Gao, Jingyi Sun, Yuting Xiao, Lu Wang, Xuan Wang, Fuyin Qu, Ming Liu, Yi Wang, Chao Gao, Jun Wang, Fengpeng Wu

**Affiliations:** Department of Radiation Oncology, the Fourth Hospital of Hebei Medical University, Shijiazhuang, Hebei, China

**Keywords:** locally advanced rectal cancer, recurrence location, radiotherapy target area, involved-field irradiation, total neoadjuvant therapy

## Abstract

**Purpose:**

This study aims to optimize neoadjuvant radiotherapy target area for locally advanced rectal cancer (LARC) patients undergoing total neoadjuvant therapy (TNT) by examining local recurrence patterns.

**Methods and materials:**

We retrospectively analyzed the clinical data of rectal cancer patients who undergone local recurrence after surgery. Recurrence sites were categorized and compared with initial diagnosis imaging, focusing on visible and suspicious lesions.

**Results:**

Of the 126 patients who met our criteria, 186 lesions were analyzed. Within these, 75.40% of cases (95/126) and 83.33% of lesions (155/186) were located within the pelvic cavity. Conversely, 3.97% of cases (5/126) and 3.33% of lesions (6/186) occurred outside the pelvic cavity. Additionally, 20.63% of cases (26/126) and 13.44% of lesions (25/186) were found in both regions. Recurrences were predominantly observed in mesenteric regions (MR) (40.86%, 76/186) and presacral regions (PR) (32.26%, 60/186). In addition, 86.51% of patients (109/126) had recurrent lesions in HRA and the suspected lesions areas. Further analysis showed that initial CEA levels and adjuvant therapy types were identified as independent predictors for recurrence in MR/PR and initially suspected lesions. 86.51% of patients had recurrent lesions in HRA and the suspected lesions areas

**Conclusion:**

The MR, PR, and areas of initial suspicious lesions are high-risk zones for post-surgical recurrence of LARC. Exploratory study of involved-field irradiation (IFI) can be carried out in the context of TNT in LARC.

## Introduction

The approach to neoadjuvant therapy for locally advanced rectal cancer (LARC) has entered the era of total neoadjuvant treatment (TNT) after went through the stages of single-chemotherapy, conventional fractionated radiotherapy alone, short-course radiotherapy (SCRT) and long-course chemoradiotherapy (LCRT). From the preferred recommendation of TNT in the National Comprehensive Cancer Network (NCCN) guideline for LARC patients, we can find that the chemotherapy regimen of CAPEOX or FOLFOX, traditionally administered postoperatively, has been arranged before the total mesorectal excision (TME), while LCRT or SCRT regimen unchanged. This shift underscores an enhanced emphasis on chemotherapy in the neoadjuvant treatment for LARC.

Radiation therapy (RT) has been a fundamental component of neoadjuvant treatment for LARC, contributing significantly to the improvement of anal preservation rate and the reduction of local recurrence rate. The delineation of neoadjuvant radiotherapy targets, historically based on Roels et al.’s 2006 analysis of postoperative recurrence sites in rectal cancer patients, did not distinguish between pre- and postoperative radiotherapy settings (1). In 2012, Valentin and his colleagues put forth guidelines for preoperative radiotherapy targeting based in varying T and N stages, later solidified in expert consensus whether the irradiation extents for lateral lymph nodes and inclusion of the ischiorectal fossa should be defined as a target (2, 3). Up to now, in clinical practice, the radiotherapy target setting of both LCRT and SCRT adheres to this consensus.

In the context of TNT mode, neoadjuvant therapy for rectal cancer has greatly increased the weight of chemotherapy. This raises pertinent questions: Will the elective nodal irradiation (ENI) of pelvic field combined with involved-field irradiation (IFI) of increasing dose in high-risk areas lead to an over-treatment? Could the successful IFI strategies used in lung and esophageal cancers be replicated in radiotherapy for LARC? Although there are no definitive answers to these questions, there is no doubt that investigating these concerns holds substantial clinical significance.

In this study, we evaluated 126 rectal cancer patients who experienced local recurrence post-surgery and analyzed the recurrence site and patterns, aiming to inform the optimal preoperative radiotherapy target setting for LARC patients under TNT mode.

## Materials and methods

### Patients

From January 2009 to July 2023, rectal cancer patients who underwent radical surgery in our hospital and were diagnosed with local recurrence during follow-up were included in this study. The main inclusion criteria were as follows: (1) Diagnosis of rectal cancer, irrespective of gender and age; (2) Complete diagnostic and treatment history in our hospital; (3) Underwent at least one abdominopelvic enhanced CT or MRI at the initial visit; (4) Underwent at least one abdominopelvic enhanced CT or MRI+DWI at the time of local recurrence diagnosis; (5) Pathological confirmation for patient who underwent surgery for local recurrent lesions; (6) Significant reduction in lesion volume and/or symptom relief after radiotherapy and/or chemotherapy in patient who did not undergo surgery. The exclusion criteria included: (1) Patients with a second primary malignancy at initial diagnosis or during follow-up; (2) Patients with both local recurrent lesions and distant organ metastasis; (3) Patients who refuse treatment for recurrent lesions; (4) Patients who do not meet the above inclusion criteria. All participants were identified in an institutional tumor registry through a protocol approved by the institutional review board with waiver of informed consent.

### Assessment of local recurrence

Local recurrence was identified by imaging as invasive or asymmetric masses not attributable to postoperative structural changes, or masses visible initially but not meeting diagnostic criteria, which then exhibited growth during follow-up. Lesions confirmed by pathological biopsy after resection or shrunk after RT and/or drug therapy were clinically diagnosed as local recurrence. The above non-pathological assessments were independently performed by two senior radiologists.

### Definition of local recurrence site

In this study, recurrence sites were classified into internal pelvic cavity (IPC) and external pelvic cavity (EPC). IPC encompassed the mesenteric regions (MR) (incl. anastomosis and rectal stump), presacral regions (PR) (Defined as: lesions located in front of the sacrum and the distance between the posterior margin of lesion and the anterior margin of the sacrum was within 1 cm), and the lateral lymphatic drainage region (LLDR) (incl. obturator, internal iliac, and external iliac). EPC included the perianal, the inguinal area (IA), and the paravascular area (PA) between the inferior mesenteric artery and the common iliac artery.

### Relationship between recurrences and initial lesions

Here, we compared and analyzed the locations of recurrent and initial lesions (incl. confirmed and/or suspicious) to identify patterns between them. A multivariate analysis was conducted to determine factors influencing site consistency.

### Statistical analysis

Statistical analysis was performed using SPSS software 25.0 (SPSS, Inc., Chicago, IL, USA) and MedCalc Version 32.0. Chi-square test was used to evaluate the distribution of recurrence sites. A forward stepwise logistic regression was used to analyze factors associated with recurrence site consistency with initial lesions. The Hosmer and Lemeshow test was used to assess the logistic regression model’s goodness of fit, and receiver operating characteristic (ROC) curve analysis was used to evaluate the model’s predictive performance. In this study, P<0.05 was considered statistically significant.

## Results

### Characteristics of enrolled patients

**Figure 1:**
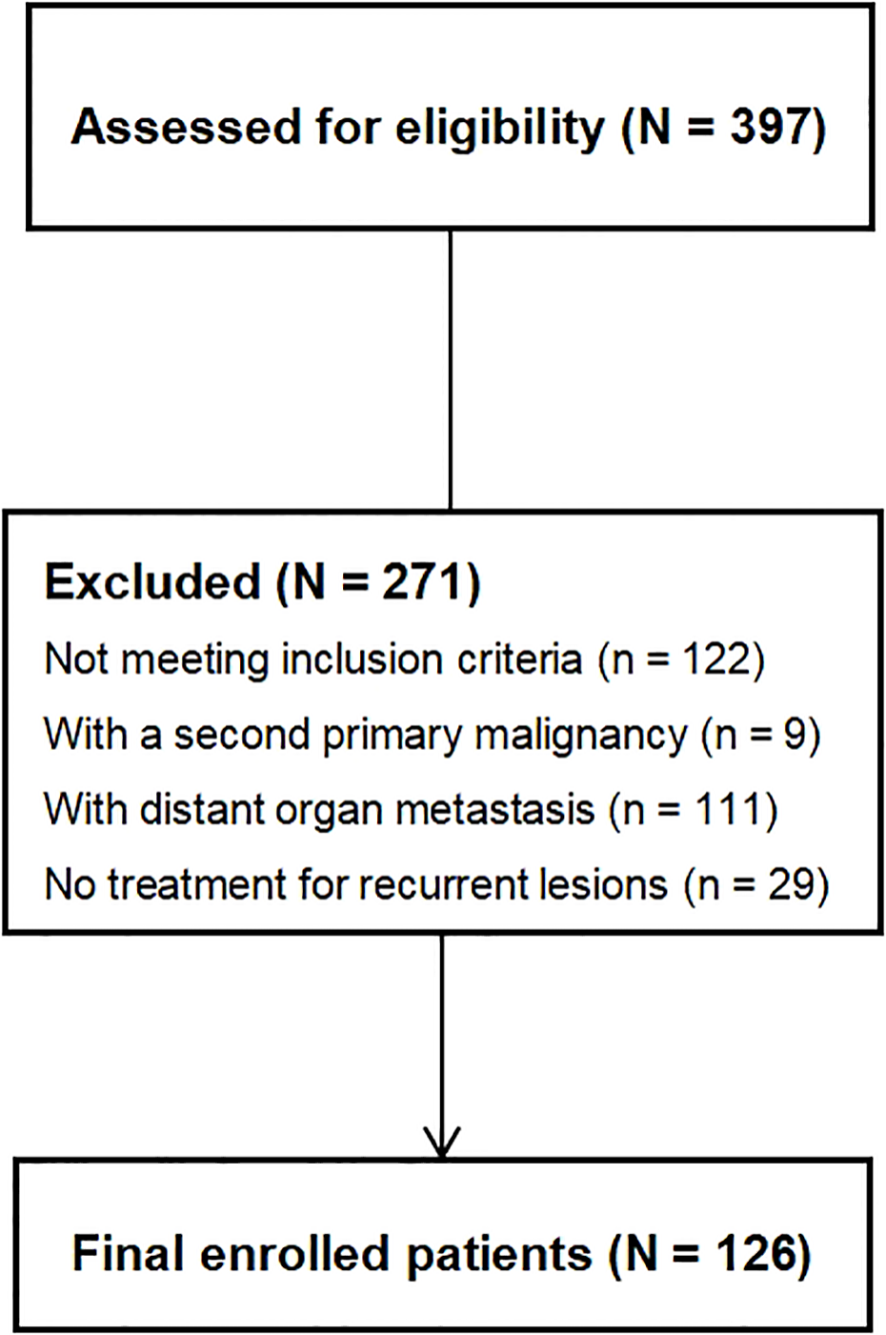
Patients selection process.

According to the inclusion and exclusion criteria, a total of 126 patients were selected for this study, as shown in **Figure 1**. Among them, 10 cases were confirmed by postoperative pathology, while 116 cases were validated based on the efficacy of nonoperative treatment. The baseline characteristics of patients are shown in **Table 1**.

**Table 1:** Baseline characteristics of patients.

Items	Characteristics	N (%)
**Gender**	Male	85 (67.46)
	Female	41 (32.54)
**Age (years)**	<30	1 (0.79)
	30-60	60 (47.62)
	≥60	65 (51.59)
**Initial CEA value (ng/ml)**	<5	58 (46.03)
	5-10	27 (21.43)
	10-20	22 (17.46)
	20-30	5 (3.97)
	30-40	3 (2.38)
	40-50	4 (3.17)
	≥50	7 (5.56)
**Distance between the lower margin of the tumor and the anal margin (cm)**	≤3	29 (23.02)
	3-5	30 (23.81)
	5-10	54 (42.86)
	>10	13 (10.32)
**Surgical methods**	Miles	44 (34.92)
	Dixon	70 (55.56)
	Hartamann	12 (9.52)
**Lateral pelvic lymphadenectomy**	no	123 (97.62)
	yes	3 (2.38)
**Pathological type**	Adenocarcinoma	98 (77.78)
	Mucinous carcinoma	28 (22.22)
**CRM**	negative	104 (82.54)
	positive	22 (17.46)
**Blood vessel invasion**	negative	112 (88.89)
	positive	14 (11.11)
**Nerve invasion**	negative	103 (81.75)
	positive	23 (18.25)
**Pathological T staging**	pT1	3 (2.38)
	pT2	16 (12.70)
	pT3	62 (49.21)
	pT4	45 (35.71)
**Pathological N staging**	pN0	53 (42.06)
	pN1	37 (29.37)
	pN2	36 (28.57)
**Preoperative adjuvant therapy**	no	109 (86.51)
	Chemotherapy alone	4 (3.17)
	chemotherapy+target therapy	0 (0)
	chemotherapy+radiotherapy	13 (10.32)
	chemotherapy+target therapy+radiotherapy	0 (0)
**Postoperative adjuvant therapy**	no	45 (35.71)
	Chemotherapy alone	52 (41.70)
	chemotherapy+target therapy	4 (3.17)
	chemotherapy+radiotherapy	24 (19.05)
	chemotherapy+target therapy+radiotherapy	1 (0.79)
**Perioperative adjuvant therapy**	no	36 (28.57)
	Chemotherapy alone	49 (38.89)
	chemotherapy+target therapy	4 (3.18)
	chemotherapy+radiotherapy	36 (28.57)
	chemotherapy+target therapy+radiotherapy	1 (0.79)
**Recurrence period (months)**	≤12	38 (30.16)
	12-24	43 (34.13)
	24-36	26 (20.63)
	36-48	9 (7.14)
	48-60	2 (1.59)
	>60	8 (6.35)
**Treatment of recurrent lesions**	Operation	6 (4.76)
	Drug therapy	30 (23.81)
	Radiotherapy	10 (7.94)
	Radiotherapy combined with drug therapy	74 (58.73)
	Drug therapy combined with operation	2 (1.59)
	Radiotherapy combined with drug therapy and operation	4 (3.17)

### Local recurrence sites

Among the 126 patients, 95 cases (75.40%) underwent IPC recurrence, with the breakdown as follows: MR (42, 33.33%), PR (28, 22.22%), MR+PR (13, 10.32%), LLDR (3, 2.38%), and combined LLDR with MR and/or PR (9, 7.14%). There were 5 cases (3.97%) of EPC recurred, including perianal (3, 2.38%), perianal and IA (1, 0.79%), PA (1, 0.79%). Additionally, 26 cases (20.63%) had both IPC and EPC recurrences, detailed in **Table 2**. In this study, all PR lesions were below the anterior inferior margin of the 2nd sacrum, and no external iliac lesions were observed in patients with LLDR recurrence. Of the 186 recurrent lesions in total, MR and PR were the most common recurrence sites, accounting for 40.86% (76/186) and 32.26% (60/186), respectively. In addition, the recurrence rates for LLDR, IA, perianal, and PA were 10.22%, 9.14%, 3.76%, and 3.76%, respectively, as shown in **Table 3**. Distribution of recurrence lesions was obviously unbalanced, which embodied that the occurrence frequency of lesions in MP and PR was much higher than that in other areas. Due to their anatomical proximity, MR and PR were collectively defined as high-frequency recurrence area (HRA) for this study.

**Table 2:** Location of local recurrent lesions of rectal cancer patients.

Location	N (%)
Internal pelvic cavity	95 (75.40)
MR	42 (33.33)
PR	28 (22.22)
MR and PR	13 (10.32)
LLDR (obturator)	3 (2.38)
MR+LLDR (obturator)	2 (1.59)
MR+LLDR (internal iliac)	2 (1.59)
PR+LLDR (internal iliac)	1 (0.79)
PR+LLDR (obturator+internal iliac)	2 (1.59)
MR+PR+LLDR (obturator)	1 (0.79)
MR+PR+LLDR (internal iliac)	1 (0.79)
External pelvic cavity	5 (3.97)
perianal	3 (2.38)
perianal+IA	1 (0.79)
PA	1 (0.79)
Internal pelvic cavity and external pelvic cavity	26 (20.63)
MR+IA	3 (2.38)
MR+PA	1 (0.79)
perianal+PR	1 (0.79)
PR+IA	7 (5.56)
PR+PA	2 (1.59)
MR+PR+IA	1 (0.79)
MR+PR+LLDR (internal iliac)+IA	1 (0.79)
MR+PR+PA	2 (1.59)
MR+PR+perianal+IA	1 (0.79)
MR+LLDR (internal iliac+obturator)	1 (0.79)
MR+LLDR (internal iliac)+PA	1 (0.79)
MR+LLDR (internal iliac+obturator)+IA	1 (0.79)
PR+LLDR (obturator+internal iliac)+IA	1 (0.79)
PR+LLDR (internal iliac)+PA	1(0.79)
PR+perianal+IA	1(0.79)
LLDR (obturator)+PA	1(0.79)

MR, mesenteric regions; PR, presacral regions; LLDR, lateral lymphatic drainage region; IA, inguinal area; PA, paravascular area between the inferior mesenteric artery and the common iliac artery.

**Table 3:** Distribution difference of local recurrent focus after radical resection of rectal cancer.

Items	Location						X^2^	P
	MR	PR	LLDR	IA	perianal	PA		
Observed (N)	76	60	19	17	7	7	140.58	0.000
Expected (N)	31	31	31	31	31	31		
Residual	45	29	-12	-14	-24	-24		

MR, mesenteric regions; PR, presacral regions; LLDR, lateral lymphatic drainage region; IA, inguinal area; PA, paravascular area between the inferior mesenteric artery and the common iliac artery.

### Pattern analysis of local recurrence sites

**Figure 2:**
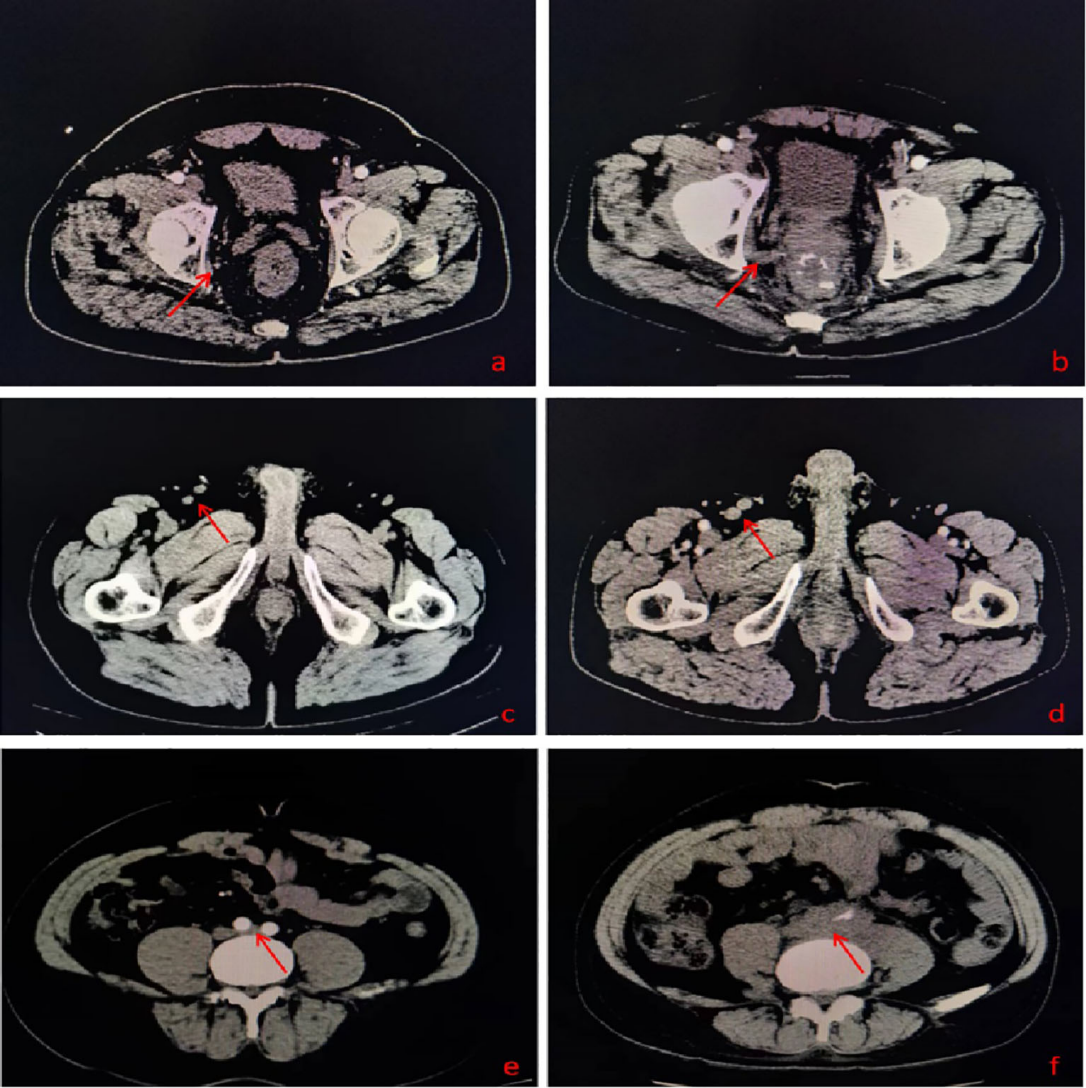
**(A)** A suspected lesion in the obturator lymphatic drainage region in initial imaging; **(B)** Recurrent lesion in the obturator lymphatic drainage region; **(C)** A suspected lesion in the inguinal lymphatic drainage region in initial imaging; **(D)** Recurrent lesion in the inguinal lymphatic drainage region; **(E)** A suspected lesion in the para-aortic lymphatic drainage region in initial imaging; **(F)** Recurrent lesion in the para-aortic lymphatic drainage region.

Comparing the initial and recurrence imaging data, we found that in addition to 83 patients with the recurrent lesions located in HRA, 26 cases’ lesions located in areas suspected in initial imaging (Incl. 9 cases in LLDR, 8 in IA, 4 in PA, 2 in LLDR+IA, 1 in LLDR+PA, and 2 in IA+PA), as shown in **Figure 2** and **Supplementary Table 1**. Beyond the 109 patients (86.51%) mentioned above, 17 (13.49%) patients had with new lesion locations not identified in initial imaging, with the breakdown as follows: 3 cases with simple perianal recurrence, 1 case with perianal+IA, 2 cases with MR+PR+perianal+IA, 2 cases with simple obturator, 1 case with MR+PR+obturator, 2 cases with MR+PR+obturator+internal iliac, 1 case with MR+PR+internal iliac, 1 case with simple IA, 1 case with IA+internal iliac, 1 case with simple PA, and 2 cases with PA+internal iliac recurrence.

Among the 186 locally recurrent lesions, 136 lesions were located in HRA, and 31 lesions located in areas suspected in initial imaging (Incl. 12 in LLDR, 12 in IA, 7 in PA), as shown in **Supplementary Table 2**. In addition to the 167 (90.76%) lesions mentioned above, 19 (10.22%) new lesions were not identified in the initial imaging, including 7 in LLDR, 5 in IA, and 7 lesions in perianal region.

**Figure 3:**
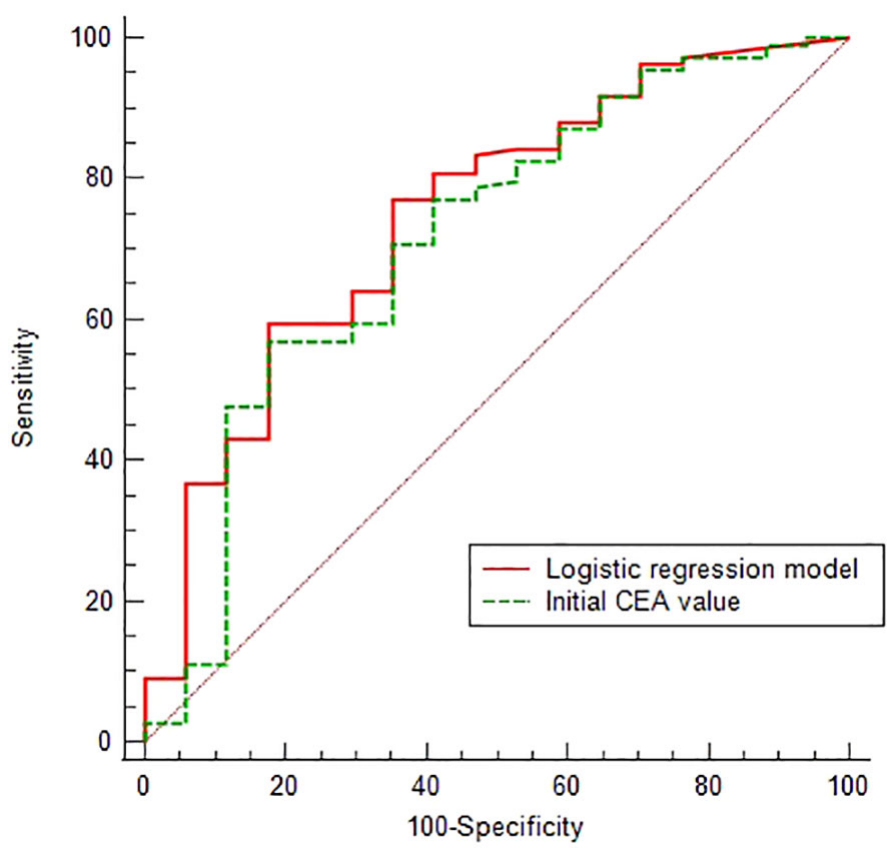
ROC analysis of initial CEA value and Logistic regression model.

Given that 86.51% of patients had recurrent lesions in HRA and the suspected lesions areas, we conducted a Logistic analysis of the relationship between 16 clinical factors and this condition (the variable assignment were detailed in **Supplementary Table 3**). In order to reduce the distortion of model evaluation, we conducted collinearity statistics for all factors before performing the logistic analysis. A variance inflation factor (VIF) of predictors ≥10, including the postoperative and perioperative treatment methods, was thought to be highly correlated with at least one of the other predictors in the aforementioned model. When excluding the factor of postoperative treatment methods from the model, we observed that the VIF of all factors was <2. Then the logistic analysis identified initial CEA values and perioperative treatment methods as a negative and a positive predictor, respectively (**Table 4**), and the Logistic regression model was $Z=sigmoid\left(X\right)=\frac{1}{1+e^{1.946-0.457X3+0.907X15}}$ . The predictive performances of the CEA and Logistic regression model were obtained by ROC analysis, yielding the Area Under Curve (AUC) of 0.713 (95% CI 0.626-0.790) and 0.747 (95% CI 0.661-0.820), respectively. On the basis of the optimal cut-off values of 6.54 ng/ml and 0.534, the sensitivity, specificity, positive predictive value and negative predictive values were 59.63% and 56.88%, 82.35% and 82.35%, 95.59% and 95.38%, 24.13% and 22.95%, respectively (**Figure 3**).

**Table 4:** Multivariate analysis of consistency between the location of recurrent lesions and the initial lesions.

Variable	B	SE	Wald	Exp(B)	P	95%CI
**Initial CEA value**	-0.456	0.139	10.688	0.634	0.001	0.482-0.833
**Perioperative treatment methods**	0.907	0.415	4.784	2.477	0.029	1.099-5.586
**Constant**	1.939	0.469	17.062	6.954	0.000	

## Discussion

With the advancement of radiotherapy technology and the renewal of treatment concept, the radiotherapy of malignant tumors has entered an era of precision. On the premise of ensuring the curative effect of patients, reasonable minimize of irradiation field is of great value in reducing radiotherapy-related damage and ensuring smooth treatment execution. This approach is evident in the shift from ENI to IFI in treating esophageal and lung cancer (4–9). As a local treatment modality, RT plays an vital role in preventing local recurrence of tumors. Consequently, it is crucial to obtain the pattern and regularity of local recurrence sites for for setting accurate radiotherapy targets.

For the recurrence site of rectal cancer, Roels et al., in their comprehensive analysis of 17 studies, reported higher local recurrence rates in the mesorectal and presacral site, at 87% and 49%, respectively (1). Our study aligns with these findings: 95 of the 126 patients had recurrent lesions located in the pelvic cavity, of which 87.37% (83/95) occurred in MR and/or PR, and 73.12% of the 186 recurrent lesions occurred in these two areas. These results underscore the MR and PR as high-risk recurrence areas in rectal cancer, warranting their inclusion in neoadjuvant radiotherapy target areas.

In clinical practice, lymph node properties of rectal cancer are generally evaluated from three aspects: whether its short diameter is greater than 0.5cm, whether the margin is regular and whether the signal is uniform. Although the introduction of the new PET functional probe of ^Ga^68 PSMA-11 and the application of magnetic resonance lymphography have significantly improved the diagnostic accuracy, widespread application of these new technologies still requires a time and experience (10, 11). Therefore, the study of determining the properties of lymph nodes by imaging technology is a research focus. In this study, 10.22%, 9.14%, and 3.76% of 186 recurrent lesions were found in the LLDR, IA and PA, respectively. Among them, 63.16% (12/19), 70.59% (12/17) and 100% (7/7) of the recurrent lesions showed suspicious lesions at the corresponding locations on the initial imaging, while new recurrent lesions in these areas only accounted for 6.45% (12/186). Therefore, we believe that strengthening the intensity of imaging examination in initial patients and incorporating suspicious lesions into the preoperative radiotherapy target could potentially reduce the local recurrence in rectal cancer patients.

As an important tumor biomarker, CEA has been widely recognized for its significant value in evaluating the disease progression and prognosis of colorectal cancer patients (12, 13). In this study, we further analyzed factors that may influence the high rate of recurrence in HRA and suspected lesion areas and found that the initial CEA value was a negative predictor of this status. Analysis of its predictive value indicated that when the cut-off value was less than or equal to 6.54 ng/ml, its positive predictive value and negative predictive value were 95.59% and 24.13%, respectively, which further indicated that low initial CEA (≤6.54 ng/ml) correlate with less aggressive tumor behavior. Additionally, we also observed that undergoing multiple perioperative treatment methods positively impacted the recurrence lesion location, emphasizing the important of intensive perioperative treatment in inhibiting tumor migration. However, the predictive model for the recurrent lesions location constructed in this study, despite its high positive predictive value (95.38%), exhibited a low negative prediction value (22.95%). Therefore, we thought that this model had limited efficacy in predicting whether new lesions would occur in the non-HRA and the sites outside the initial suspected lesion area.

At present, the TNT mode is the only preoperative regimen recommended by the NCCN guidelines for LARC patients with pMMR/MSS status (14). This regimen marks a significant shift from the traditional LCRT and SCRT, primarily by intensifying the chemotherapy component to a duration of 12-16 weeks. Previous studies have underscored that pivotal role of chemotherapy in the neoadjuvant setting for rectal cancer. For example, the EORTC 22921 trial involving 1011 patients demonstrated that adding chemotherapy to preoperative radiotherapy in cT3-4 resectable rectal cancer patients enhanced tumor downstaging and downsizing (15), with patients showing ypT0-2 status gaining benefits in disease-free survival and overall survival (16). Maas and colleagues analyzed 3313 patients from 13 datasets based on their response to neoadjuvant chemoradiotherapy (nCRT) and found that although adding chemotherapy during the interval between radiotherapy and surgery did not benefit the prognosis of patients who obtained pathological complete response (pCR), it was beneficial for patients with ypT1-2 and ypT3-4 (17). In the recent PROSPECT trial, researchers even mentioned the concept of omitting radiotherapy in neoadjuvant therapy of LARC patients, although they recruited patients with only cT1-3 and not anxious about preserving sphincter. This idea further underscores the growing importance of chemotherapy in treatment (18).

While the impact of pCR on long-term prognosis remains a subject of debate (19–22), it is clear that neoadjuvant chemoradiotherapy offers high-response patients the opportunity for anal preservation, low recurrence, and adoption of “*Wait and Watch*”. In the exploration of LCRT based TNT mode, Garcia-Aguilar and colleagues found that patients receiving sequential 6-cycle mFOLFOX6 following nCRT exhibited a pCR rate more than double that of those who received only nCRT (38% vs.18%) (23). Similarly, in the SCRT-based TNT model, the RAPIDO Trial reported significantly higher pCR rates with the SCRT sequential 6-cycle CapeOX or 9-cycle FOLFOX4 regimen compared to nCRT (28.37% vs. 14.32%) (24, 25). These results highlight that the increased weight of chemotherapy in neoadjuvant therapy significantly improves the responsiveness of LARC patients to neoadjuvant therapy.

According to the requirements of ICRU 83 (26), the target area of neoadjuvant radiotherapy for rectal cancer needs to include MR, PR, LLDR, and the ischiorectal fossa of patients with levator ani muscle invasion. Patients undergoing long-course nCRT also need to implement additional irradiation on the MR and the affected lymph drainage area, that is, a combination of ENI and IFI. In addition, previous research had demonstrated that intensified radiotherapy directed at the primary tumor (not on the lymph nodes) could also enhance tumor regression rates, attain higher pCR rate and reduce the local recurrence rate. Especially for patients with difficulty in preserving anus or with high-risk factors (MRF+, T4, etc.) (27–29).

At present, there are still few studies on IFI in the target setting of pelvic malignancies. YANG et al. analyzed the prognostic factors affecting patients with recurrent ovarian cancer and found that the use of ENI or IFI target setting mode did not lead to differences in survival (30). Li et al. compared elderly bladder cancer patients receiving IFI and ENI, noting no significant difference in overall survival and local progression-free survival between the two groups. However, the acute toxicity rate in the IFI group was significantly lower than that in the ENI group (45.23% vs 72.00%, P=0.008) (31). In our study, a substantial majority of patients (86.51%, 109/126) and relapses (90.86%, 167/186) occurred in HAR and regions with preoperatively suspicious positive lymph nodes. Therefore, we believe that exploring IFI for LARC patients in the context of TNT treatment has important clinical value.

Although we obtained complete data on local recurrence in patients with rectal cancer, our retrospective analysis spanning 14 years has limitations. Firstly, to ensure the completeness of the clinical data, this study only reviewed 126 patients in our center and did not conduct a multi-center review. Secondly, in the analysis of factors that affect the location of recurrent lesions in HRA and suspicious lesion locations, due to the complexity of patient treatment plans and courses, we only assigned orderly values to the treatment methods. While our analysis indicated a higher correlation of recurrence location with intensive treatment, further research is needed to validate these findings, particularly in the context of IFI. Thirdly, we did not perform internal or external validation on the predictive model due to a limited number of patients with new recurrent lesions outside HRA and suspicious lesions. In the future, we will continue to accumulate data or conduct multi-center reviews to complete this work.

In summary, this study classified the sites of recurrent lesions of rectal cancer and compared them with the initial images, and preliminarily discovered the patterns of recurrence sites. Based on this, we believe that exploring IFI for LARC patients is feasible, especially in the context of TNT mode. Should IFI demonstrate similar efficacy and prognosis to ENI, it could significantly reduce radiotherapy adverse events and treatment delay in LARC patients.

## Data Availability

The original contributions presented in the study are included in the article/**Supplementary Material**. Further inquiries can be directed to the corresponding author.
