# Supplementary material for: Could elective nodal irradiation for locally advanced rectal cancer be omitted in the context of total neoadjuvant therapy? An analysis of the recurrence sites of rectal cancer
Source: Front Oncol. 2024 Nov 27;14:1459024. doi: 10.3389/fonc.2024.1459024 (PMC11631729; doi:10.3389/fonc.2024.1459024)
Supplement: Supplementary file 1 [file Table1.docx]

Supplementary Table 1 The situation of patients with recurrence located in HRA and suspected lesion areas at initial diagnosis

| Location | N (%) |
| --- | --- |
| HRA | 83 (65.87) |
| HRA+LLDR | 9 (7.14) |
| HRA+IA | 8 (6.35) |
| HRA+PA | 4 (3.17) |
| HRA+LLDR+IA | 2 (1.59) |
| HRA+LLDR+PA | 1 (0.79) |
| HRA+IA+PA | 2 (1.59) |
| Total | 109 (86.51) |

**Note:** HRA=MR+PR; LLDR=lateral lymphatic drainage region; IA=inguinal area; PA=paravascular area between the inferior mesenteric artery and the common iliac artery
