# Supplementary material for: Could elective nodal irradiation for locally advanced rectal cancer be omitted in the context of total neoadjuvant therapy? An analysis of the recurrence sites of rectal cancer
Source: Front Oncol. 2024 Nov 27;14:1459024. doi: 10.3389/fonc.2024.1459024 (PMC11631729; doi:10.3389/fonc.2024.1459024)
Supplement: Supplementary file 2 [file Table2.docx]

Supplementary Table 2 The situation of recurrent lesions located in the HRA and initially suspected lesion areas

| Location | N (%) |
| --- | --- |
| HRA | 136 (73.91) |
| LLDR | 12 (6.52) |
| IA | 12 (6.52) |
| PA | 7 (3.80) |
| Total | 167 (90.76) |

**Note:** HRA=MR+PR; LLDR=lateral lymphatic drainage region; IA=inguinal area; PA=paravascular area between the inferior mesenteric artery and the common iliac artery
