# Supplementary material for: Could elective nodal irradiation for locally advanced rectal cancer be omitted in the context of total neoadjuvant therapy? An analysis of the recurrence sites of rectal cancer
Source: Front Oncol. 2024 Nov 27;14:1459024. doi: 10.3389/fonc.2024.1459024 (PMC11631729; doi:10.3389/fonc.2024.1459024)
Supplement: Supplementary file 3 [file Table3.docx]

Supplementary Table 3 Stratification and assignment of variables in Logistic regression analysis

| Items | Variable | Layering and Assignment |
| --- | --- | --- |
| Y | Recurrent lesions located in OM+AS and/or initial suspicious site | no=0; yes=1 |
| X1 | Gender | male=0; female=1 |
| X2 | Age (years) | <30=0; 30-60=1; ≥60=2 |
| X3 | Initial CEA value (ng/ml) | <5=0; 5-10=1; 10-20=2; 20-30=3; 30-40=4; 40-50=5; ≥50=6 |
| X4 | Distance between the lower margin of the tumor and the anal margin (cm) | ≤3=0; 3-5=1; 5-10=2; >10=3 |
| X5 | Surgical methods | anal-preserving operation=0; non anal preservation surgery=1 |
| X6 | Lateral pelvic lymphadenectomy | no=0; yes=1 |
| X7 | Pathological type | adenocarcinoma=0; mucinous carcinoma=1 |
| X8 | CRM | negative=0; positive=1 |
| X9 | Blood vessel invasion | negative=0; positive=1 |
| X10 | Nerve invasion | negative=0; positive=1 |
| X11 | T staging | T1=0; T2=1; T3=2; T4=3 |
| X12 | N staging | N0=0; N1=1; N2=2 |
| X13 | Preoperative treatment methods | no=0; systemic therapy=1; systemic therapy+radiotherapy=2 |
| X14 | Postoperative treatment methods | no=0; systemic therapy=1; systemic therapy+radiotherapy=2 |
| X15 | Perioperative treatment methods | no=0; systemic therapy=1; systemic therapy+radiotherapy=2 |
| X16 | Recurrence period (months) | <12=0; 12-24=1; 24-36=2; 36-48=3; 48-60=4; ≥60=5 |
